# How I manage ICP-CPP: a visual, yet individualized approach

**DOI:** 10.1186/s13054-019-2565-8

**Published:** 2019-08-27

**Authors:** William D. Freeman

**Affiliations:** 1 Department of Neurology, Mayo Clinic, 4500 San Pablo Rd, Jacksonville, FL 32224 USA; 2 Department of Neurosurgery, Mayo Clinic, Jacksonville, FL USA; 3 Department of Critical Care Medicine, Mayo Clinic, Jacksonville, FL USA

I read with great interest Drs. Robba and Citerio’s [1] approach to intracranial pressure (ICP)-cerebral perfusion pressure (CPP) management, and it is to be commended. My approach over the years has evolved to teach a visual *pyramidal* approach to our nurses, residents, fellows, and now our advanced practice providers and neurosurgeons. Rather than use the Tier 0, 1, 2, 3 system as proposed by the Neurocritical Care Society in Emergency Neurologic Life Support, I often simply provide this Fig. 1 to our teams to show the foundation is laid with basics of CPP (mean arterial pressure-ICP) management. This visual diagram shows that to measure CPP, an ICP monitor and basic interventions like head/neck positioning are needed. The diagram also demonstrates the importance of emphasizing the ICP-CPP zero at the tragus for standardization [2, 3]. These fundamentals cannot be overstated, especially with nurses eager to re-emphasize at bedside the goals of care of the patient. Further, beyond basic CPP management, osmotherapy comes into play, which once exhausted, moves up the pyramid to *escalation* therapies of refractory ICP, including barbiturates or hypothermia, and ultimately to neurosurgical decompression (“top of the pyramid” literally and figuratively). We find this Fig. 1 useful for discussion, and even management with our fellows, as well as for long-standing issues about use of mannitol versus say hypertonic saline in osmotherapy selection, etc. We find that there is an insatiable academic thirst for knowledge around this topic each year among all team members and hope this Fig. 1 provides food for thought for similar teams at other centers [4].

**Fig. 1:**
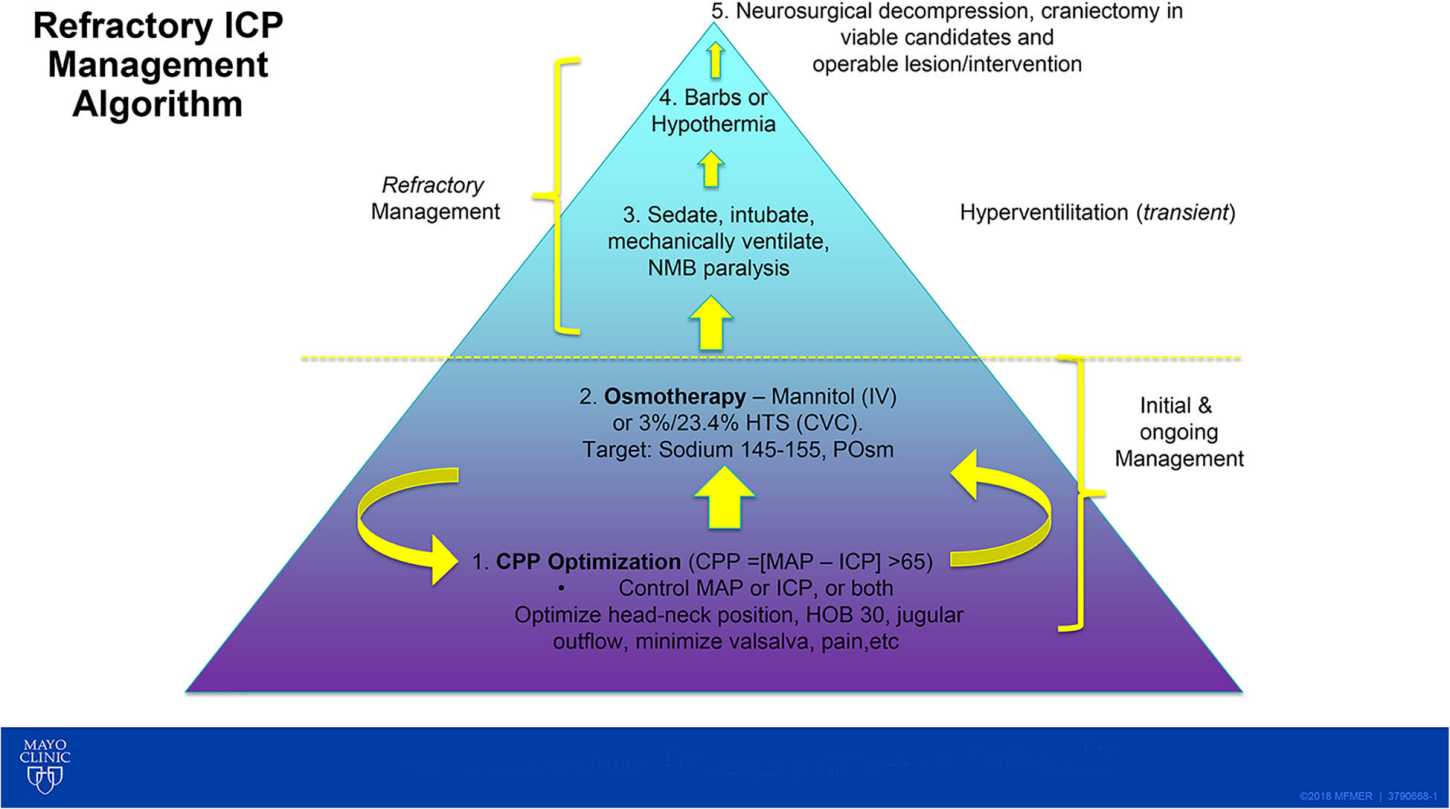
Pyramidal approach to ICP-CPP management. Barbs indicates barbiturates; CPP, cerebral perfusion pressure; CVC, central venous line; HOB, head of bed; HTS, hypertonic saline; ICP, intracranial pressure; IV, intravenous; MAP, mean arterial pressure; NMB, neuromuscular blockade; POsm, plasma osmolality. Used with permission of Mayo Foundation for Medical Education and Research, all rights reserved

## Data Availability

NA.
